# Real‐world experience of paediatric acute promyelocytic leukaemia in the United Kingdom and Ireland

**DOI:** 10.1111/bjh.19843

**Published:** 2024-11-17

**Authors:** Aditi Vedi, Sarah Maria Leiter, Irum Latif Memon, Arunthethy Mahendrayogam, Elsje Van Rijswijk, Urmila Uparkar, Geoff Shenton, Amelie Trinquand, Katherine Clesham, Vanessa McLelland, Helen Campbell, Katharine Patrick, Lyndsey Thompson, Susan Baird, Philip Connor, Donna Lancaster, Beki James

**Affiliations:** ^1^ Cambridge University Hospitals NHS Foundation Trust Cambridge UK; ^2^ Department of Paediatrics University of Cambridge Cambridge UK; ^3^ Great Ormond Street Hospital London UK; ^4^ Royal Marsden Hospital Surrey UK; ^5^ Newcastle Hospitals NHS Foundation Trust Newcastle UK; ^6^ National Children's Cancer Service Children's Health Ireland at Crumlin Dublin Ireland; ^7^ University College London Hospital London UK; ^8^ University Hospitals Bristol NHS Foundation Trust Bristol UK; ^9^ Manchester University Hospitals Manchester UK; ^10^ Sheffield Children's NHS Foundation Trust Sheffield UK; ^11^ Royal Belfast Hospital for Sick Children Belfast UK; ^12^ Royal Hospital for Children and Young People Edinburgh UK; ^13^ Health Care Research Wales Cardiff UK; ^14^ Leeds Children's Hospital Leeds UK

**Keywords:** all‐trans retinoic acid, APL, arsenic trioxide, Leukaemia, Paediatric

## Abstract

Acute promyelocytic leukaemia (APL), defined by the t(15;17)(q24;q21) translocation, accounts for 5%–10% of paediatric acute myeloid leukaemia cases. All‐trans retinoic acid (ATRA) and arsenic trioxide (ATO) are key treatments, though ATO access varies. We evaluated treatment, complications and survival in 50 UK paediatric APL patients diagnosed between 2014 and 2021. All patients received ATRA and most received ATO. Event‐free survival was lower in high‐risk patients (85% vs. 100%, *p* = 0.03), and those not receiving ATO at diagnosis. All relapsed patients could be salvaged with ATO. Addressing ATO availability and consistent funding is crucial to ensure timely treatment and improve outcomes.

## INTRODUCTION

Acute promyelocytic leukaemia (APL) is a rare subtype of acute myeloid leukaemia (AML) characterised by distinct molecular abnormalities, unique clinical features and requiring specific treatment strategies. It accounts for 5%–10% of paediatric AML, amounting to six to eight cases annually in the United Kingdom. Coagulopathy at presentation is the commonest cause of death. Risk stratification is based on white cell count (WCC): WCC ≥10 × 10^9^/L at diagnosis classed as high risk (HR) and WCC <10 × 10^9^/L as standard risk (SR).

The presence of the t(15;17)(q24;q21) chromosomal translocation results in fusion of the promyelocytic leukaemia (*PML*) and retinoic acid receptor alpha (*RARA*) genes, and formation of the PML–RARA fusion protein. This plays a pivotal role in leukaemogenesis^1^ and is a key therapeutic target. All‐trans retinoic acid (ATRA), a vitamin A derivative, promotes the degradation of the fusion protein and restores normal myeloid differentiation, inducing complete remission in most APL patients.^2^, ^3^ Arsenic trioxide (ATO), a potent anti‐leukaemic agent, is also an effective therapeutic agent for APL.^4^, ^5^ Other fusions may also lead to APL, which are still sensitive to ATRA.^6^

Combining ATO with ATRA has significantly improved outcomes for patients with APL, enabling delivery of a chemotherapy‐free regimen for most patients.^4^, ^5^, ^7^ However, access to ATO has been limited by funding constraints in some countries. We present real‐world data describing treatment, funding, complications and survival of all paediatric patients diagnosed with APL in the United Kingdom and the Republic of Ireland (ROI) over 7 years.

## METHODS

### Data collection

In this retrospective descriptive study, children aged 0–18 years with newly diagnosed APL presenting between 01 November 2014 and 31 October 2021 were identified through a national survey. Demographics, treatment, complications and outcome were collected in a pro forma.

During this period, there were no national paediatric treatment guidelines for APL in the United Kingdom. Patients were treated with variable combinations of ATRA, ATO, immunotherapy and chemotherapy, based on the ICC‐APL‐01 trial^2^ and adult data.^4^

### Statistical analysis

Progression‐free survival (PFS) and overall survival (OS) were evaluated. PFS and OS were defined as time from diagnosis to relapse or disease progression, or death by any cause respectively. All direct comparisons between SR and HR groups were performed using Fisher's exact test. All statistical analyses and data visualisations were performed in R (www.r‐project.org), and tables generated using BioRender.

## RESULTS

### Demographics

Fifty patients aged 1–18 years (median 12 years) were diagnosed with APL in the United Kingdom and ROI across 14 of 22 paediatric haematology centres during the study period (Table 1). There was equal sex distribution; all patients had bone marrow (BM) disease at diagnosis and three patients had extramedullary involvement (CNS, skin and gingival). There were 21 (42%) HR patients and 29 (58%) SR patients. The median age was higher in the SR group (median 13.7 years vs. 11 years, *p* = 0.03, Figure S1). Three patients presented with intracranial haemorrhage (ICH) peri‐diagnosis.

**TABLE 1:** Demographics of patients <18 years diagnosed with APL in the UK and Ireland over a 7‐year period (1 November 2013 to 31 October 2021), and drugs used in treatment of APL patients.

Demographics	SR = 29	HR = 21	Total = 50
Sex	15 male (48%)	9 male (43%)	24 male (48%)
	14 female (52%)	12 female (57%)	26 female (52%)
Median age	12 y 5 mo (2y 11 mo to 18 y 6 mo)	11 y 9 mo (1 y 3 mo to 17 y)	12 y 4 mo (1 y 3 mo to 18 y 6 mo)
Country	25 England	17 England	42 England
	2 N Ireland	3 ROI	2 N Ireland
	1 ROI	1 Wales	4 ROI
	1 Scotland		1 Scotland
			1 Wales
Site of disease	29 BM	21 BM	50 BM
	1 skin	1 CNS	1 CNS
	1 gingiva		1 skin
			1 gingiva
ICH at diagnosis	2 ICH	1 ICH	3 ICH

Treatment	SR	HR	Total
ATRA	29/29 (100%)	20/21 (95%)	49/50 (98%)
ATO	26/29 (90%)	18/21 (86%)	44/50 (88%)
Idarubicin	829 (67%)	14/21 (67%)	22/50 (44%)
Gemtuzumab ozogamicin	0/29 (0%)	3/21 (14%)	3/50 (6%)
Mitoxantrone + cytarabine	3/29 (10%)	0/21 (0%)	3/50 (6%)
Cytarabine + daunorubicin + etoposide	0/29 (0%)	2/21 (9%)	2/50 (4%)
Mitoxantrone	1/29 (3%)	0/21 (0%)	1/50 (2%)
6MP + MTX	0/29 (0%)	1/21 (4%)	1/50 (2%)

Abbreviations: 6MP, 6‐mercaptopurine; ATO, arsenic trioxide; ATRA, all‐trans retinoic acid; BM, bone marrow; CNS, central nervous system; HR, high risk; ICH, intracranial haemorrhage; mo, month; MTX, methotrexate; ROI, Republic of Ireland; SR, standard risk; y, year.

### Treatment

All patients received ATRA at diagnosis, except one who died prior to receiving any treatment. Most patients also received ATO: 85.7% and 89.6% in the HR and SR groups respectively. Some patients also received cytotoxic agents, idarubicin being the most common, with 62% vs. 27.5% receiving this in the HR and SR groups respectively.

Ten patients received other cytotoxic agents: six (2 HR and 4 SR) in induction and one in maintenance. Three HR patients at a single centre received gemtuzumab ozogamicin (GO) in addition to ATRA and ATO at induction.

Two HR patients received induction treatment with cytarabine, daunorubicin and etoposide (ADE) only, with one patient having ATRA added on day 12 of induction to a delay in APL diagnosis; and another patient, diagnosed outside the United Kingdom, receiving ATRA and ATO in course 2 after arrival in the United Kingdom. Among the four SR patients, three patients received mitoxantrone and cytarabine (MA) induction, one of whom had a delayed diagnosis of APL, and another had mitoxantrone added in cycle 2 due to poor compliance with oral ATRA during induction. One HR patient received 6‐mercaptopurine and methotrexate as maintenance treatment.

Three patients presented with severe ICH peri‐diagnosis and required emergency decompression craniotomy. Of these, one died prior to receiving any systemic therapy, one received ATRA only and died 4 days after diagnosis, and the third patient is in CR1.

### Supportive care

Fifteen patients in the cohort (30%) received hydroxycarbamide as supportive therapy, of whom five were HR and 10 were SR. Thirty‐three patients received preventative corticosteroids as either prophylaxis or treatment for differentiation syndrome: 71% of HR and 62% of SR patients.

### Adverse events

The distribution of complications was similar between HR and SR groups (Figure S2) and included differentiation syndrome, pseudotumour cerebri and headache. Survival was not affected by the presence of complications during treatment (Figure S3).

### Treatment funding source

**FIGURE 1:**
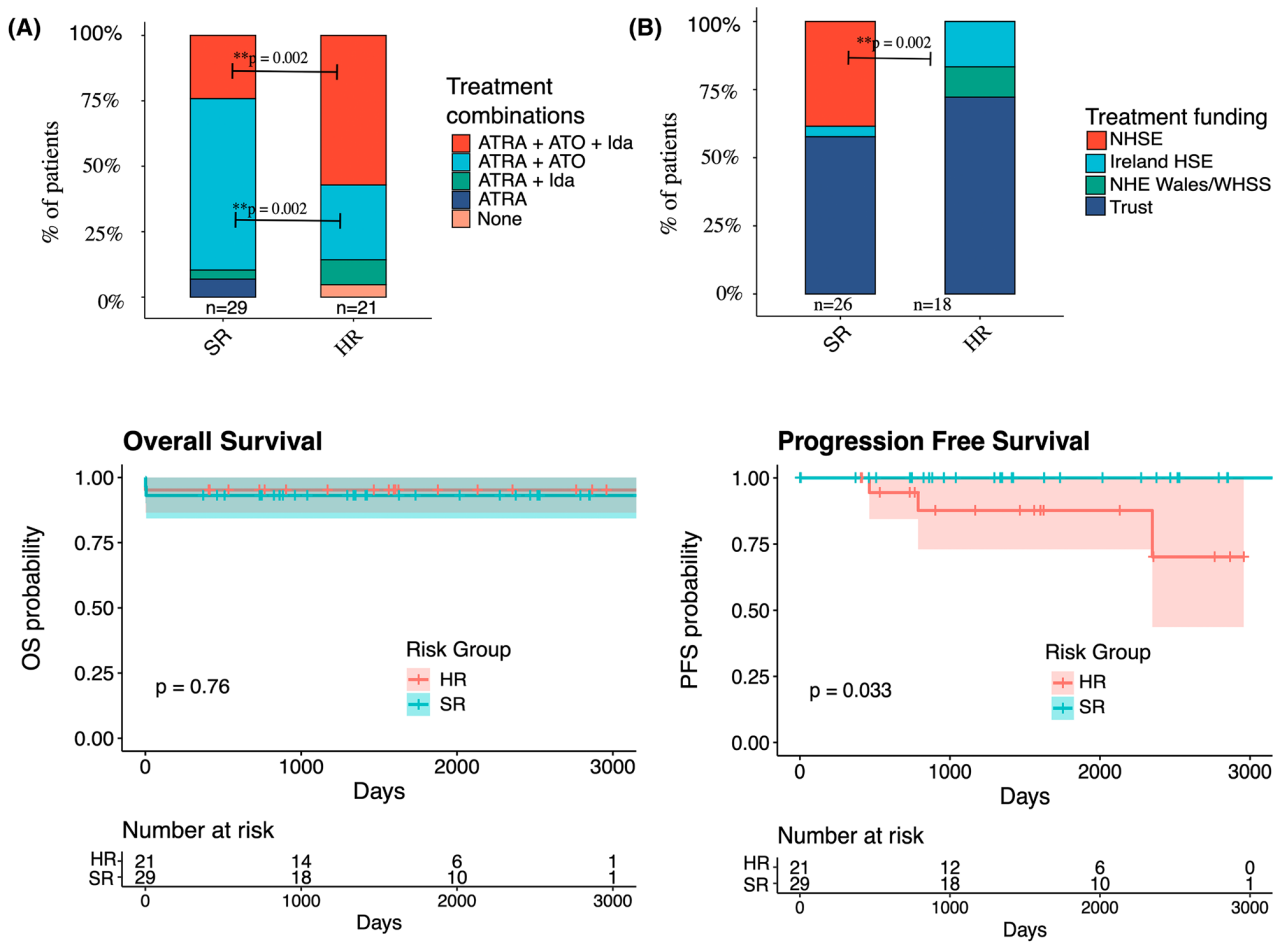
Treatment combinations, funding source for ATO and survival of paediatric APL in the United Kingdom, 2014–2021. (A) Bar chart representing treatment combinations used to treat non‐HR and HR patients at diagnosis in this cohort. (B) Bar chart representing source of funding for ATO for SR and HR patients. *p*‐values calculated using Fisher's exact test. (C) Kaplan–Meier survival curves for patients by risk group in this cohort. (Left) Overall survival, (Right) Progression‐free survival. ATO, arsenic trioxide; ATRA, all‐trans retinoic acid; HR, high risk; Ida, Idarubicin; SR, standard risk.

ATRA and other treatment were locally funded by individual NHS Trusts across the United Kingdom, while funding for ATO varied. Of the 44 patients receiving ATO, 15 (34%) received central funding (Figure 1B). In England, ATO was funded locally by the individual NHS Trust in 25/36 cases (70%) and centrally by NHSE in 11/36 cases (30%). Two patients with HR disease in England did not receive ATO due to lack of funding. All Northern Irish and Scottish patients in this cohort received treatment funded by their local trust, while all Welsh and ROI patients received central funding for their treatment. Of the three SR patients who did not receive ATO, all were from England, two were treated with ATRA alone and one received ATRA and Idarubicin.

### Survival

Survival data are derived from minimum 2‐year follow‐up from diagnosis, with median follow‐up of 8 years (Figure 1C). The OS was 94% (47/50 patients) in this cohort, with no statistically significant difference between HR and SR patients (*p* = 0.76). Two patients (1 HR and 1 SR) died due to ICH, one prior to receiving systemic therapy for APL. There was another early death in the SR group, related to differentiation syndrome on day 2 of treatment. PFS was significantly lower in HR versus SR patients, 85% vs. 100% at 8 years (*p* = 0.03).

OS was significantly higher in patients receiving ATO (44/44 vs. 3/6; 100% vs. 50%, *p* < 0.0001). PFS was also higher, but without reaching statistical significance, in patients receiving ATO at diagnosis (95% vs. 66%, Figure S4). Source of funding for ATO did not significantly alter survival (Figure S5).

### Relapse/refractory treatment

Three patients relapsed (Table S1), all HR, of whom two had ATO + ATRA and another had ATRA and Idarubicin at diagnosis; the latter patient experienced late relapse 6.5 years from diagnosis. Median time to relapse was 3 years 4 months from diagnosis. Relapse treatment was universally with ATO + ATRA, with two patients receiving Cytarabine in addition and one also receiving GO. All three relapsed patients are alive in CR2.

One SR patient had refractory disease (MRD positive) following induction (ATRA + idarubicin) and three courses of consolidation (ATRA, cytarabine, mitoxantrone and idarubicin), but responded to ATO + ATRA, achieving CR1 almost 12 months from initial diagnosis.

Treatment, complications and survival for all 50 patients in the cohort are summarised in Figure S6.

## DISCUSSION

This is the largest single cohort of paediatric patients treated with combination therapy, ATRA + ATO, describing real‐world experience of access to both drugs, adverse effects and outcomes. Overall, the survival data from this cohort describe excellent outcomes for APL with the universal use of ATRA.^2^, ^4^, ^7^

Mortality occurred early in diagnosis and was related to coagulopathy, specifically ICH and differentiation syndrome. OS was comparable with previous studies with and without ATO.^2^, ^5^ However, within this cohort patients receiving ATO had improved OS. It is not clear whether delays in accessing ATO contributed to disease progression and death from acute complications. PFS was also very high, consistent with other studies. Importantly, PFS was higher in the group of patients receiving ATO at diagnosis irrespective of risk group. All relapsed patients were salvaged with ATO + ATRA, even if both drugs had been used at initial diagnosis. Two patients also received idarubicin and one received GO at relapse. The addition of GO upfront for HR patients is currently being evaluated in the ICC APL Study 02 (NCT04793919).

The addition of ATO to the treatment of all APL patients is now standard of care, and its tolerability is acceptable.^5^, ^8^ Combined ATRA and ATO treatment allows omission of maintenance treatment as per the COG AAML 1331 study.^8^ Here, we further demonstrate safety and tolerability of the combination regimen of ATO + ATRA. Treatment‐related toxicity was mainly associated with ATRA, with one patient requiring cessation due to pseudotumour cerebri. No ATO‐related toxicity was reported.

Access to ATO is not currently universal within the United Kingdom. In practice, funding is mostly, but not always, absorbed by local NHS trusts with a new funding request per patient, introducing a potentially significant delay in treatment, to the detriment of their overall outcome. This real‐world experience of paediatric APL has led to a policy proposition paper currently under consideration with NHSE to enable access to ATO for all risk groups. A standardised guideline including universal ATRA and ATO treatment has since been developed by the Children's Cancer and Leukaemia Group in the United Kingdom.

## AUTHOR CONTRIBUTIONS

AV designed the data collection pro forma, collated all data, performed all analyses and wrote the manuscript. SML critically revised the paper and provided data visualisation in Figure S6. BJ designed the research question, provided oversight and critically appraised the paper and all analyses. DL provided national guidance and oversight and critically appraised the paper. All other authors provided data and critically revised the paper once written.

## FUNDING INFORMATION

No funding was received for this research.

## CONFLICT OF INTEREST STATEMENT

No conflicts of interest declared.

## ETHICS APPROVAL STATEMENT

The study did not require institutional review board (IRB) approval as it was an audit. Audits are exempt from needing IRB approvals in the United Kingdom as they are a means of ensuring that patients are being treated optimally according to the guideline. This research was endorsed by the Children's Cancer and Leukaemia Group, specifically the Childhood Leukaemia Clinician's Network subgroup (with DL as chair of this group).

## PATIENT CONSENT STATEMENT

Written consent was obtained for treatment.

## Supporting information


Data S1.

## Data Availability

All relevant clinical data are included within the supplementary materials.
