# Supplementary material for: Real‐world experience of paediatric acute promyelocytic leukaemia in the United Kingdom and Ireland
Source: Br J Haematol. 2024 Nov 17;206(1):204–8. doi: 10.1111/bjh.19843 (PMC11739768; doi:10.1111/bjh.19843)

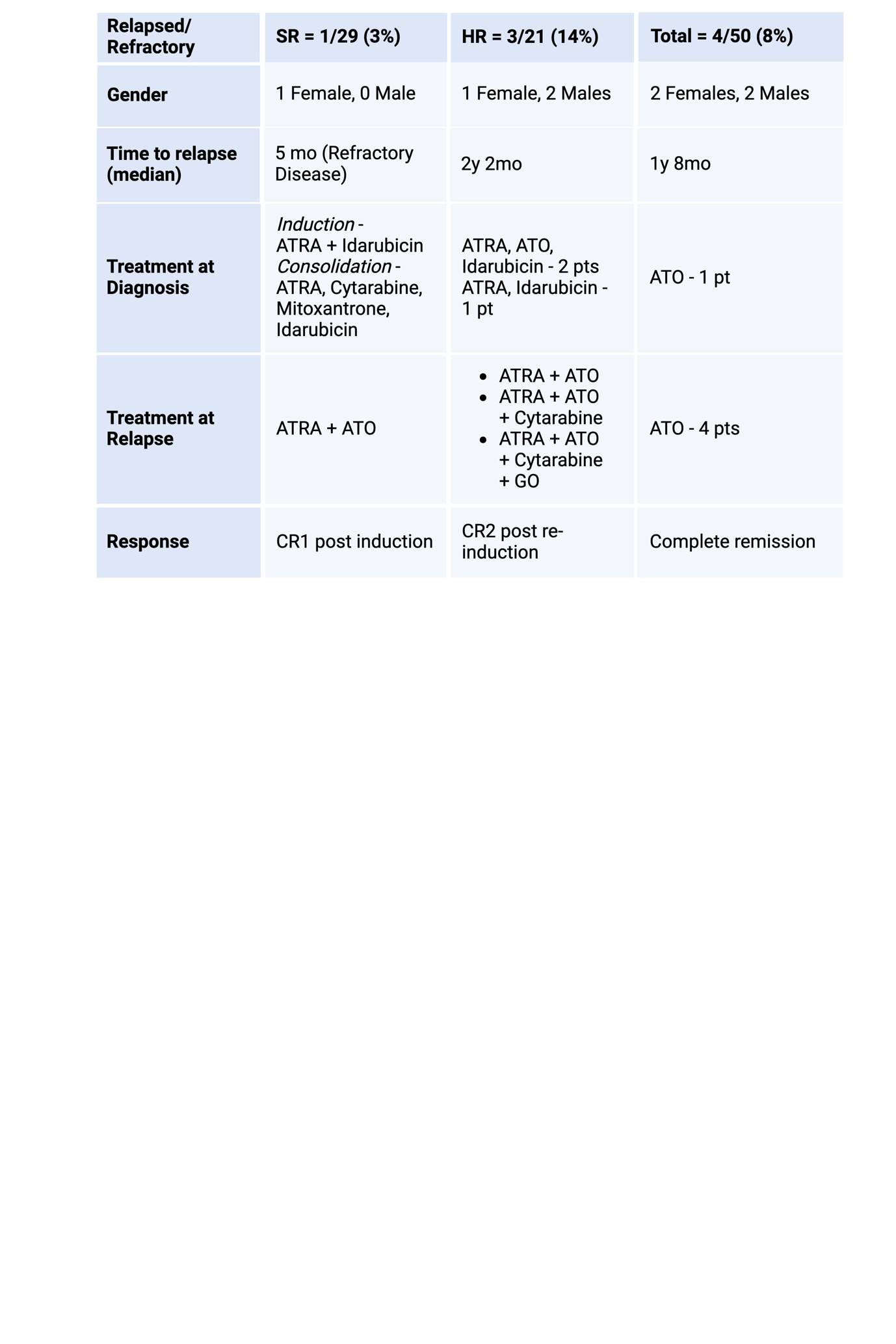


**Supp Table 1:** patient characteristics and treatment used at relapse. Total 4 patients in the cohort.

**Supplementary Figures**

Supp Fig1: Age range and median age at diagnosis of HR and Non-HR children with APL.

Supp Fig2: Frequency of complications during treatment between risk groups.

Supp Fig3: OS and PFS in all patients with or without differentiation syndrome

Supp Fig4: OS and PFS related to source of treatment funding

Supp Fig5: OS and OFS related to use of ATO

Supp Fig6: Treatment, complications and survival of paediatric APL in the UK, 2014 -2021. Heatmap showing the distribution of treatments, complications of diagnosis and treatment, and survival (progression or death) amongst the entire cohort. Abbreviations: ATO = arsenic trioxide, ATRA = all-trans retinoic acid, MA = mitoxantrone + cytarabine, GO = gemtuzumab ozogamicin, G2 = Grade 2.


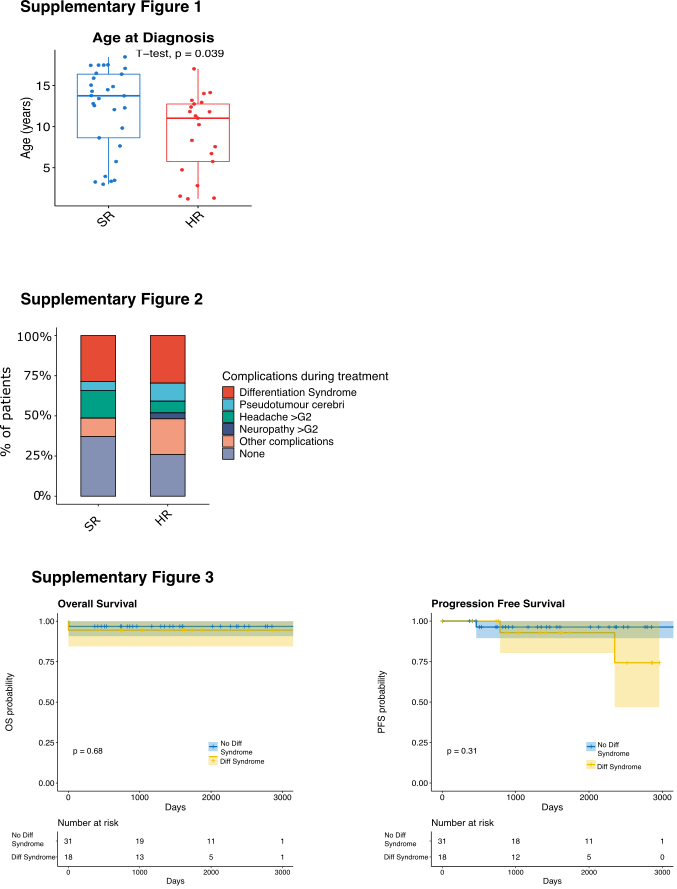


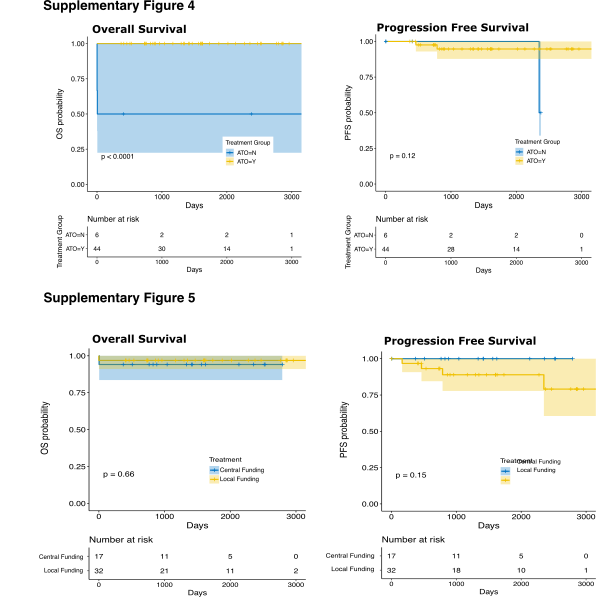

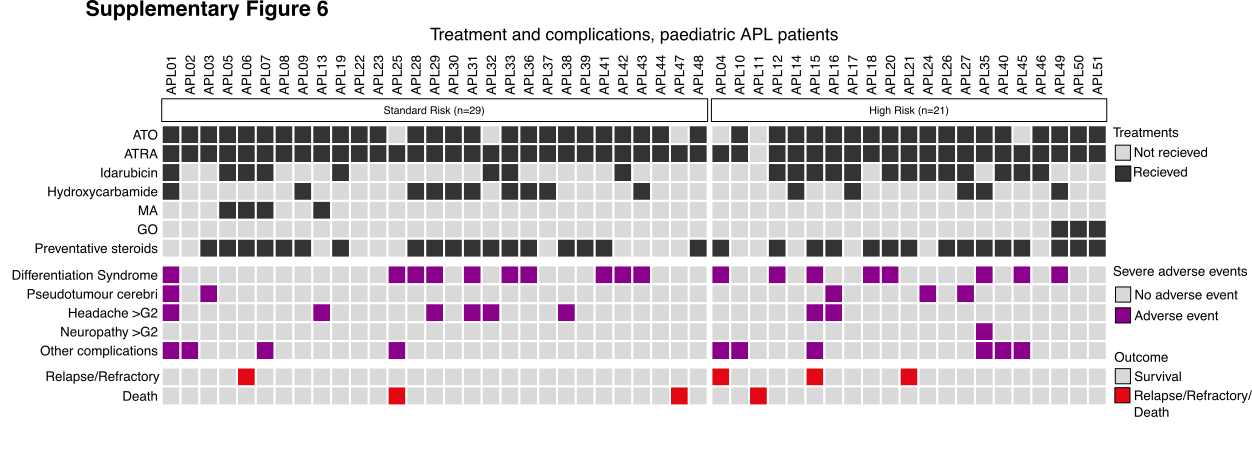

Supplement: Supplementary file 1 — Data S1. [file BJH-206-204-s001.docx]
